# The DNA Methylation Inhibitor Zebularine Controls CD4^+^ T Cell Mediated Intraocular Inflammation

**DOI:** 10.3389/fimmu.2019.01950

**Published:** 2019-08-16

**Authors:** Yanli Zou, Xiao Hu, Lauren P. Schewitz-Bowers, Madeleine Stimpson, Li Miao, Xiaofei Ge, Liu Yang, Yan Li, Paul W. Bible, Xiaofeng Wen, Jing Jing Li, Yizhi Liu, Richard W. J. Lee, Lai Wei

**Affiliations:** ^1^State Key Laboratory of Ophthalmology, Zhongshan Ophthalmic Center, Sun Yat-sen University, Guangzhou, China; ^2^Translational Health Sciences, University of Bristol, Bristol, United Kingdom; ^3^National Institute for Health Research Biomedical Research Centre at Moorfields Eye Hospital NHS Foundation Trust and UCL Institute of Ophthalmology, London, United Kingdom

**Keywords:** uveitis, zebularine, DNA methylation inhibitor, CD4^+^ T cell, intraocular inflammation

## Abstract

CD4^+^ T cell mediated uveitis is conventionally treated with systemic immunosuppressive agents, including corticosteroids and biologics targeting key inflammatory cytokines. However, their long-term utility is limited due to various side effects. Here, we investigated whether DNA methylation inhibitor zebularine can target CD4^+^ T cells and control intraocular inflammation. Our results showed that zebularine restrained the expression of inflammatory cytokines IFN-γ and IL-17 in both human and murine CD4^+^ T cells *in vitro*. Importantly, it also significantly alleviated intraocular inflammation and retinal tissue damage in the murine experimental autoimmune uveitis (EAU) model *in vivo*, suggesting that the DNA methylation inhibitor zebularine is a candidate new therapeutic agent for uveitis.

## Introduction

Autoimmune uveitis is a heterogeneous collection of diseases characterized by intraocular inflammation (1). Affected patients are at risk of visual impairment or blindness (2), in particular those who are resistant or intolerant to conventional immunosuppressive therapies (3–5). Consequently there is a need to develop improved approaches to achieve control of intraocular inflammation.

Abnormal activation of the T helper (Th) cells and imbalance between inflammatory Th1/Th17 and regulatory T (Treg) cells play a key role in the pathogenesis of autoimmune uveitis (6–8). Modulating Th cell differentiation and function has been proposed as a therapeutic strategy for uveitis (9). The expression of signature cytokines and master transcription factors, as well as Th functions, are under tight control of coordinated epigenetic alterations (10, 11). And many genome-wide and locus-specific epigenetic changes have been found in patients with immune-mediated diseases such as systemic lupus erythematosus and rheumatoid arthritis (12). Therefore, modulating the epigenetic program that controls Th1, Th17, and Treg functions may serve as a new way to control the intraocular inflammation in uveitis.

Two key epigenetic mechanisms—DNA methylation and histone modifications—regulate chromatin accessibility, and have been the targets of a series of compounds developed in recent years for the treatment of cancers and immune-mediated diseases (12). Among these small-molecule inhibitors, two DNA methyltransferase (DNMT) inhibitor pro-drugs, 5-azacytidine

and 2′-deoxy-5-azacytidine have been approved for the treatment of myelodysplastic syndrome and acute myeloid leukemia (13). In addition, an anti-inflammatory effect of these two drugs has also been observed in murine models of asthma (14) and experimental autoimmune encephalomyelitis (15). We therefore hypothesize that another DNA methylation inhibitor, zebularine, which has relatively low cellular toxicity and a longer half-life (16), has the potential to suppress uveitis.

Here, to elucidate whether zebularine is able to control the intraocular inflammation through regulating the expression of inflammatory cytokines in CD4^+^ T cells, we explored the changes of IFN-γ, IL-17, and Foxp3 (17) in response to zebularine treatment in Th1, Th17, and Treg cells. In addition, the immunosuppressive effects of zebularine *in vivo* were also evaluated using the murine experimental autoimmune uveitis model.

## Materials and Methods

### Human Peripheral Blood CD4^+^ T Cell Isolation

CD4^+^ T cells were obtained by negative selection from peripheral blood of healthy controls (HCs) (*N* = 4; 3 female and 1 male; average age of 42) following informed consent in accordance with National Health Service Research Ethic Committee approved protocols at the University Hospitals Bristol Foundation Trust, United Kingdom (04/Q2002/84). Written, informed consent was obtained from all study participants. CD4^+^ T cells were obtained by incubating up to 80 ml uncoagulated peripheral blood with RosetteSep™ Human CD4^+^ T cell Enrichment Cocktail (Stemcell Technologies, Canada) according to the manufacturer's instructions. Blood was then layered on Ficoll-Paque PLUS (GE Healthcare, USA) and centrifuged for 1,200 × g for 20 min. Enriched CD4^+^ T cells were removed from the density gradient and washed in RPMI-1640 (Thermofisher) supplemented with 10% fetal calf serum (FCS). The purity of human CD4^+^ T cells was >95%.

### Mice

Female C57BL/6 2-Hb mice (8–10 weeks) were purchased from Guangdong Medical Laboratory Animal Center (China) and maintained in a SPF facility in Zhongshan Ophthalmic Center of Sun Yat-sen University. All animal experiments were approved by the Institutional Animal Care and Use Committee of Zhongshan Ophthalmic Center. All animal work was performed in compliance with the ARVO Statement for the Use of Animals in Ophthalmic and Vision Research.

### Antibodies and Reagents

Zebularine was purchased from Tocris Bioscience (UK). The recombinant mouse interleukin-6 (rmIL-6), rmIL-12, recombinant human transforming growth factor beta 1 (rhTGF-β1) were purchased from R&D Systems (USA). Functional antibodies anti-CD3 (145-2C11) and anti-CD28 (37.51), FACS antibodies anti-IL-17A (eBio17B7), anti-IFN-γ (XMG1.2) and anti-Foxp3 (FJK-16s) were from eBioscience (USA). CD4 (L3T4) and naïve CD4^+^ T cell Magnetic-activated cell sorting (MACS) isolation kits were purchased from Miltenyi (Germany). The complete Freund's adjuvant and pertussis toxin were purchased from Sigma-Aldrich (USA).

### Cell Culture

Human peripheral CD4^+^CCR6^−^ and CD4^+^CCR6^+^ cells (*N* = 4; 3 female and 1 male; average age of 42) were FACS-sorted from isolated CD4^+^ T cells, resuspended to 2 × 10^6^ cells/mL in RPMI-1640 supplemented with 10% FCS, L-glutamine, and penicillin/streptomycin (all Thermofisher, stimulated with plate-bound anti-CD3 (5 μg/mL; clone UCHT1) and anti-CD28 (5 μg/mL;clone CD28.2) antibodies (eBioscience) (CCR6^−^ cells) or with plate-bound antibodies and a polarizing cytokine mixture of 20 ng/mL IL-6, 10 ng/mL IL-23, 10 ng/mL IL-1β (all from R&D Systems), 100 ng/mL anti-IFN-γ, 100 ng/mL anti-IL-4 (eBioscience) (CCR6^+^ cells) for 5 days. Zebularine was also added at the indicated concentrations at the beginning of cultures. For murine CD4^+^ T cells, spleen and lymph node cells were filtered through a 40-μm cell strainer, followed by the red blood cell lysis using ammonium-chloride-potassium (ACK) buffer. Total CD4^+^ T cells were then isolated with the CD4 (L3T4) T cell isolation kit using the autoMACS Pro Separator (Miltenyi, Germany) and stimulated by plate-bound of anti-CD3 (5 μg/mL) and anti-CD28 (2 μg/mL) antibodies, as well as mIL-12 10 ng/mL, mIL-2 10 ng/mL, anti-IL-4 10 μg/mL (Th1); mIL-6 20 ng/mL, rhTGF-β1 1 ng/mL, anti-IL-4 10 μg/mL, anti-IFN-γ 10 μg/mL (Th17); and mIL-2 50 ng/mL, rhTGF-β1 2 ng/mL (Treg) for 3 days. When zebularine was used, the indicated doses of drug were added at the beginning of the culture.

### RNA-seq Analysis

Total RNA from Th1, Th17, and Treg cells was extracted with the MasterPure Complete DNA and RNA Purification Kit (Epicentre, UK) according to the manufacture's instruction. A total of 100 ng RNA was sonicated into fragments of 300–400 base pairs using Bioruptor (Diagenode, Belgium). mRNA library was prepared using VAHTS mRNA-seq V3 Library Prep Kit for Illumina (Vazyme, Nanjing, China) following the manufacture's protocol and sequenced on the Illumina HiSeq2500 sequencer with HiSeq SR Cluster Kit V4 and HiSeq SBS Kit V4 50 cycle kit (Illumina). The initial processing was performed by CASAVA (v1.8.2). Sequencing reads were then subjected to quality control processed by FastQC (v0.11.5) and trimmed by Cutadapt (v1.9.1). Quality controlled reads were then analyzed using DEseq2.

### Quantitative Real-Time PCR (qPCR)

Total RNA from T cells was extracted with the MasterPure Complete DNA and RNA Purification Kit (Epicentre, UK), and cDNA was synthesized with the PrimeScript™ cDNA Synthesis Kit (Takara, Japan). qRT-PCR was performed using SYBR Green PCR Master Mix (KAPA Biosystems, USA) on the Light Cycler 480 instrument (Roche, Switzerland). The samples were run in duplicate and the relative expression was determined by normalizing the expression of each gene to glyceraldehyde 3-phosphate dehydrogenase (GAPDH) using the 2^−ΔΔ*Ct*^ method. The primers used are shown in the Supplementary Table 1.

### Drug Treatment in Mice

Zebularine was reconstituted with 0.9% saline to make a 1 μg/μL stock and stored at −80°C. Zebularine was administered to the mice at 10 μg/g body weight by intraperitoneal injection. The injection was started on day 7 post-immunization and performed daily for 7 consecutive days. The control group was injected with 0.9% saline only. Body weights of both groups were monitored after drug treatment.

### EAU Induction and Scoring

C57BL/6 mice were immunized subcutaneously, at the base of tail (100 μL) and in both thighs (50 μL), with total 50 μg human interphotoreceptor retinoid binding (IRBP) _651−670_ emulsified with complete Freund's adjuvant supplemented with 3.5 mg *Mycobacterium tuberculosis*. Each mouse also received 1 μg pertussis toxin (Tocris Bioscience, UK) intraperitoneally. To assess the clinical score of EAU, we performed dilated-pupil fundus examination with a Micron III murine fundus camera (Phoenix Research Labs, USA) and histological assessment of FFPE sections of the retinal tissues from EAU mice (18). The clinical scores were given by two independent experienced observers in a blindfold manner based on the criteria for EAU scoring as described previously (19).

### Flow Cytometry and FACS Sorting

For FACS-sorted human cells, pre-enriched CD4^+^ T cells were incubated with a combination of primary antibodies against cell surface markers at 4°C for 30 min and then washed using PBS (v/v 10% FCS and 1mM EDTA). Antibodies used for human CCR6 T cell FACS sorts were anti-CD4 AF700 (BD Biosciences), anti-CD3 BV510 (BD Biosciences) and CCR6 PE (BD Biosciences). 7-AAD (Biolegend, USA) was used to discriminate living cells. Cells were sorted into CD4^+^CCR6^−^ and CD4^+^CCR6^+^ subsets using a BD Influx (BD Biosciences) and routinely >95% purity was achieved. Both human and murine cultured or single cell suspensions collected from human CCR6 5-day cultures and retina, spleen, or lymph nodes from mice were stimulated with 20 ng/ml phorbol myristate acetate (PMA), 1 μM ionomycin (Sigma-Aldrich, USA) and 1 μl/ml GolgiStop (BD Biosciences) for 4 h at 37°C. After 4 h, cells were washed with PBS (v/v 10% FCS and 1mM EDTA), stained with Infrared Live/Dead fixable dye (ThermoFisher) fixed and permeabilized (Cytofix/perm solution; BD Biosciences) and stained with anti-IL-17A, anti-IFN-γ, and anti-Foxp3 antibodies. Samples were acquired using a BD LSRFortessa™ X-20 (BD Biosciences). Results were analyzed using Flowjo v10 (Treestar).

### Bisulfite Sequencing

Total DNA isolated from mouse polarized Treg cells was extracted with the MasterPure Complete DNA and RNA Purification Kit (Epicentre, UK). Sodium bisulfite treatment of total DNA was performed using the EZ DNA Methylation-Gold Kit (Zymo Research, USA) according to the manufacturer's instruction. PCR amplification was performed on the T100 Thermal Cycler (Bio-Rad, USA) in a final volume of 50 μL. PCR products were purified with the Zymoclean Gel DNA Recovery Kit (Zymo Research, USA). The sequencing results were analyzed by BiQ Analyzer (Max Planck Institut Informatik, Germany). The following primers pairs were used: Foxp3 enhancer CpG island (−5782 to −5558): (F: AATG TGGG TATT AGGT AAAA TTTT T; R: AAAC CCTA AAAC TACC TCTA AC), Foxp3 promoter CpG island (−5252 to −5030): (F: TAGG TGAT TGAT AAGT AGGA GAAG TTAG TA; R: TACC CCCA TTAC TTAT AACC ATTTC).

### Statistical Analysis

Statistical analysis was performed with Prism Graphpad 7.0 (GraphPad Software, USA). Mann-Whitney *U* test or one-way ANOVA test was used accordingly.

## Results

### Zebularine Restrains the Expression of IFN-γ and IL-17A in CD4^+^ T Cells *in vitro*

**Figure 1:**
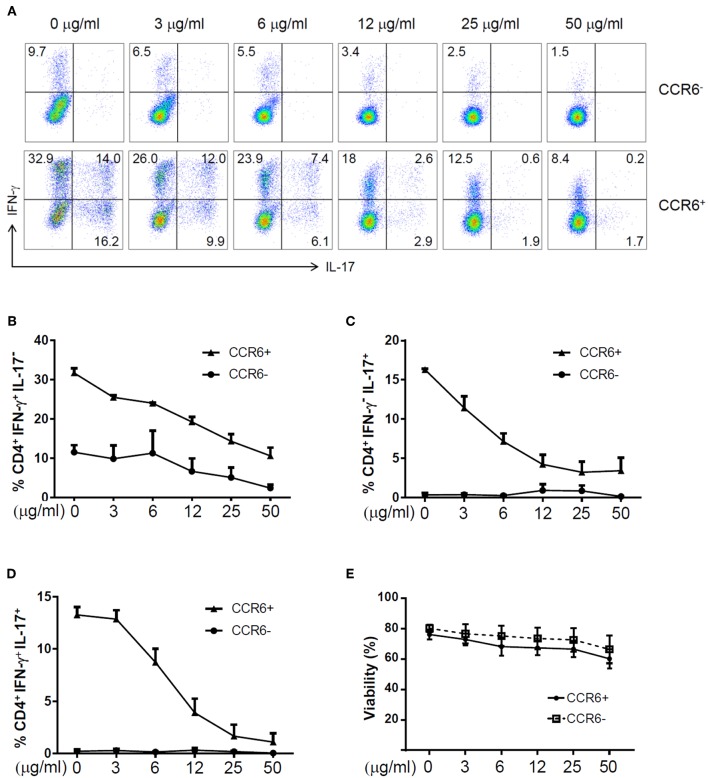
The expression of IFN-γ and IL-17A in zebularine-treated human CD4^+^ T cells *in vitro*. **(A)** Representative dot plots (*N* = 4; 3 female and 1 male; average age of 42) for stimulated human CD4^+^CCR6^−^ and CD4^+^CCR6^+^ cells on Day 5, stained with IFN-γ and IL-17A. The frequency of CD4^+^IFN-γ^+^IL-17^−^ **(B)**, CD4^+^IFN-γ^−^IL-17^+^ **(C)**, and CD4^+^IFN-γ^+^IL-17^+^ **(D)** Cells in zebularine-treated human CD4^+^CCR6^+^ and CD4^+^CCR6^−^ cells. **(E)** Viability of CD4^+^CCR6^−^ and CD4^+^CCR6^+^ cells treated with different doses of zebularine. Means ± SD are shown.

To assess the effects of zebularine on the expression of proinflammatory cytokines, we first examined the human peripheral CD4^+^ T cells. FACS-sorted CD4^+^CCR6^−^ and CD4^+^CCR6^+^ cells were stimulated with anti-CD3/anti-CD28 antibodies or under the Th17 polarizing condition, respectively. As shown in Figure 1, the frequency of IFN-γ^+^IL-17^−^ cells was significantly reduced in both CCR6^+^ and CCR6^−^ cells in response to zebularine treatment (Figures 1A,B), while IL-17 expression was restrained by zebularine in a dose dependent manner (Figures 1A,C,D). The viability of CD4^+^ T cells was not significantly affected by zebularine treatment (Figure 1E). These data suggest that zebularine is able to control the inflammatory cytokine expression in human CD4^+^ T cells.

**Figure 2:**
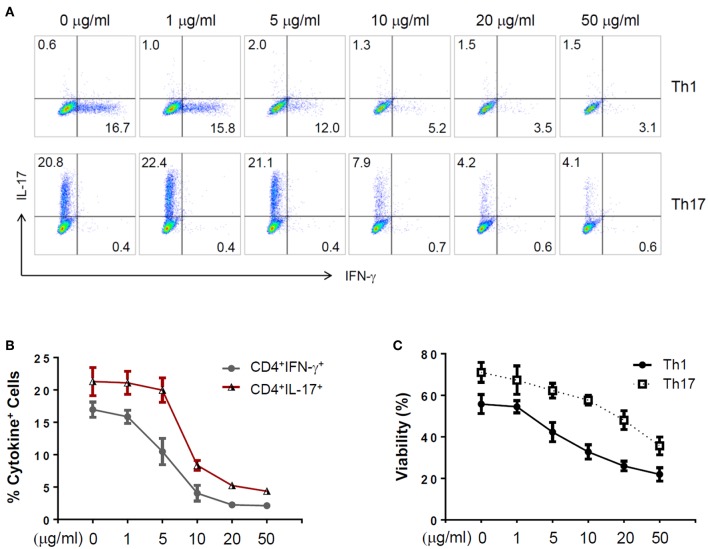
The expression of IFN-γ and IL-17A in zebularine-treated murine Th1 and Th17 cells *in vitro*. **(A)** Representative dot plots (*N* = 5) for murine Th1 and Th17 cells stained with IFN-γ and IL-17A. **(B)** The percentages of IFN-γ^+^ and IL-17^+^ cells in zebularine-treated murine CD4^+^ T cells. **(C)** Viability of murine Th1 and Th17 cells treated with different doses of zebularine. Means ± SD are shown.

**Figure 3:**
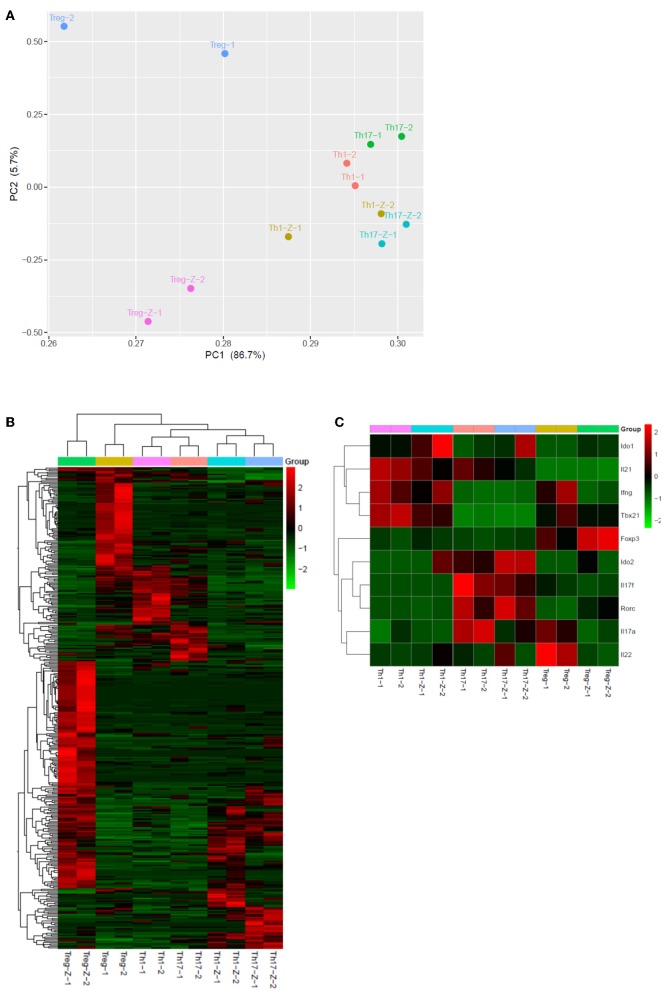
Genome-wide expression profiles of Th1, Th17, and Treg cells in response to zebularine stimulation. **(A)** Principle component analysis of genes with at least two-fold changes between any two conditions and *P* < 0.05 (one-way ANOVA). -Z represents the conditions with zebularine stimulation. **(B)** Hierarchical clustering analysis of Th1, Th17, and Treg cells with (-Z) or without zebularine stimulation. **(C)** Hierarchical clustering of genes of interest in Th1, Th17, and Treg cells.

**Figure 4:**
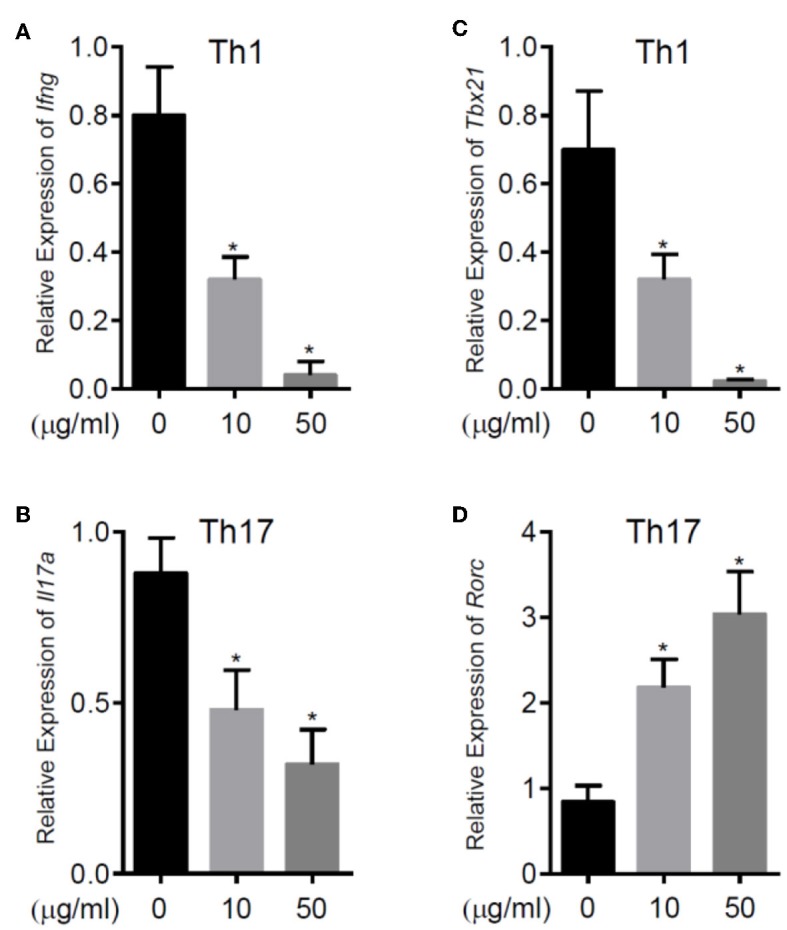
The relative RNA expression of *Ifng* **(A)** and *Tbx21* **(C)** in murine Th1 cells, *Il17a* **(B)** and *Rorc* **(D)** in murine Th17 cells. RNA was extracted from murine CD4^+^ T cell subsets polarized for 3 days with or without zebularine and evaluated by qRT-PCR. *N* = 5, *P < 0.05.

To further explore the immunomodulatory effect of zebularine in murine cells, we differentiated murine peripheral CD4^+^ T cells into inflammatory Th1 and Th17 conditions. Similar to the findings in human CD4^+^ T cells, zebularine was found to significantly suppress the protein expression of IFN-γ and IL-17A (Figures 2A,B) in murine Th1 and Th17 cells, respectively. However, the cell viability was significantly compromised in response to those high dose zebularine treatments (Figure 2C). To confirm the global changes zebularine stimulation may lead to on CD4^+^ T cells, we performed genome-wide expression analysis of murine Th1 and Th17 cells in response to zebularine stimulation using the mRNA-seq technology. The principal component analysis (Figure 3A) and hierarchical clustering analysis (Figure 3B) of all of the differentially expressed genes (defined as a two-fold change between any two conditions, with a one-way ANOVA *P* < 0.05) revealed that zebularine significantly changed global gene expression profiles of Th1 and Th17 cells, in addition to the significant modulation of *Il17a* expression in Th17 cells (Figure 3C). We next performed quantitative PCR analysis to confirm the changes of mRNA expression of the inflammatory cytokines and key transcription factor *Tbx21*, which encodes the Th1 master regulator Tbet found by RNA-seq analysis. The expression of *Ifng* (Figure 4A), *Tbx21* (Figure 4C), and *Il17a* (Figure 4B) was significantly decreased by zebularine in Th1 and Th17 cells, respectively, while the relative RNA expression of *Rorc* was increased in Th17 cells in response to zebularine treatment (Figure 4D). Taken together, our data demonstrated the immunomodulatory function of zebularine on both human and murine CD4^+^ T cells *in vitro*.

### Zebularine Promotes the Expression of Foxp3 in Murine CD4^+^ T Cells *in vitro*

**Figure 5:**
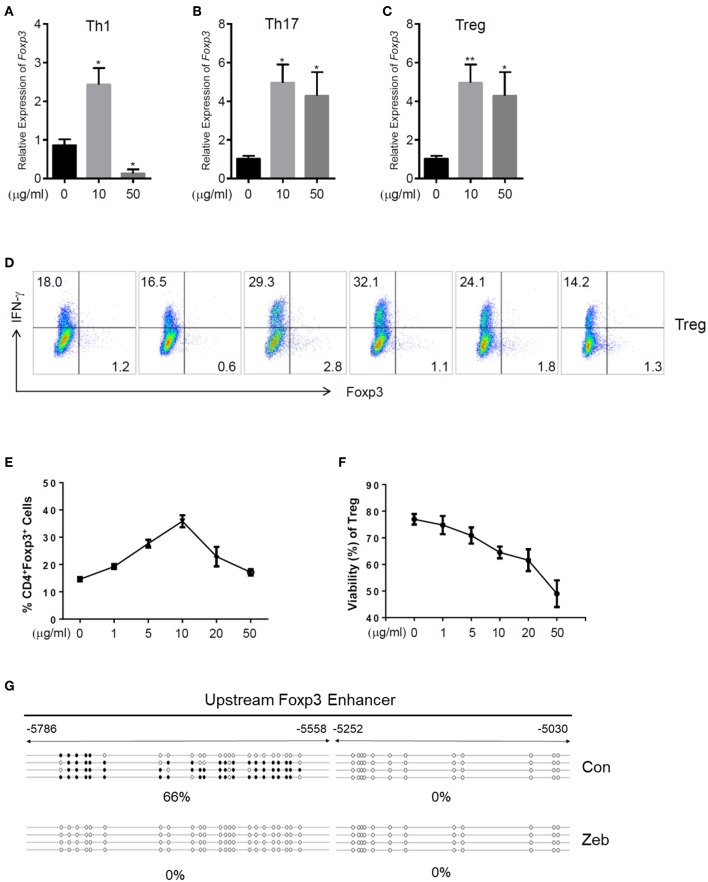
Zebularine promotes the expression of Foxp3 in murine CD4^+^ T cells *in vitro*. **(A–C)** The relative RNA expression of *Foxp3* in zebularine-treated murine Th1, Th17 and Treg cells *P < 0.05; **P < 0.01. **(D)** Representative dot plots (*N* = 5) for murine Treg cells stained with IFN-γ and Foxp3. **(E)** The percentages of Foxp3^+^ cells in zebularine-treated murine CD4^+^ T cells. Means ± SD are shown. **(F)** Viability of murine Treg cells treated with different doses of zebularine. Means ± SD are shown. **(G)** Methylation status of the CpG islands located in *Foxp3* enhancer in murine Treg cells treated with zebularine or vehicle. •, methylated CpG; ∘, unmethylated CpG.

In addition to the effects on cytokine expression, we also investigated whether zebularine modulated the expression of Foxp3, the key transcription factor controlling the development and function of Treg (20). The genome-wide expression analysis demonstrated a significant response of Treg cells to the zebularine stimulation (Figures 3A,B). As shown in Figures 3C, 5A,B, the RNA expression of *Foxp3* was activated and significantly increased in response to the treatment of zebularine in Th1 and Th17 cells. Under inducible Treg polarizing condition, high dose of zebularine was not able to further elevate the expression of Foxp3 (Figures 5C–E). It is noteworthy that zebularine treatment at 10 μg/mL significantly decreased the viability of Th1 cells (Figure 2C), while the Treg cells were still highly viable (Figure 5F). These data suggest that zebularine promotes the expression of Foxp3 in CD4^+^ T cells.

The epigenetic regulation of Foxp3 expression by DNA methylation alterations was well documented by previous studies (21). Therefore, we next assessed whether the methylation status of the CpG islands located in *Foxp3* enhancer was changed by zebularine in Treg cells, using the bisulfite sequencing technique. As shown in Figure 5G, the *Foxp3* upstream enhancer (−5786 to −5558) was hypermethylated in the vehicle-treated control cells (66% methylated), while all 23 CpG islands were demethylated in cells treated with zebularine (10 μg/mL). These data further demonstrate that zebularine promotes the expression of Foxp3 in CD4^+^ T cells through demethylating its enhancer region.

### Zebularine Controls Intraocular Inflammation *in vivo*

**Figure 6:**
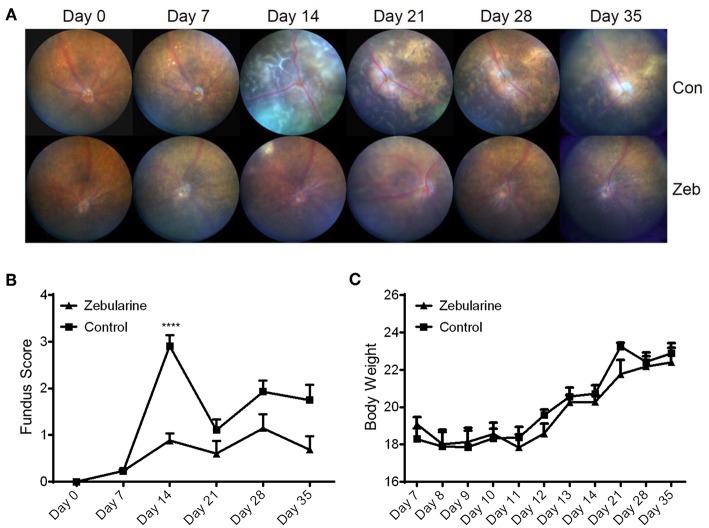
The intraocular inflammation of EAU mice was attenuated by zebularine treatment *in vivo*. The representative fundus images **(A)** and fundus scores **(B)** (means ± SD) of EAU mice treated with zebularine (*N* = 10) or vehicle (*N* = 10) were shown ****P < 0.0001. **(C)** Body weights of EAU mice (means ± SD) treated with zebularine (*N* = 10) or vehicle (*N* = 10) were monitored every day during treatment and every week after treatment.

**Figure 7:**
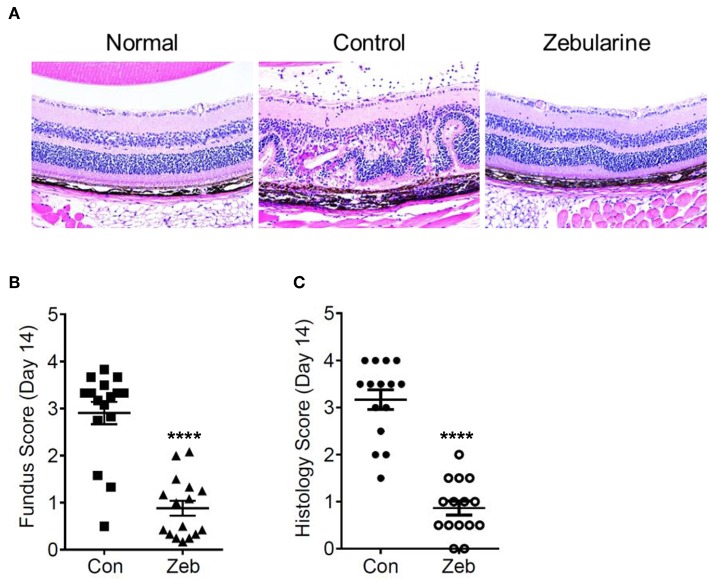
Histological analyses of the retinal tissues from EAU mice treated with zebularine or vehicle on Day 14 post immunization. **(A)** Representative HE histological sections of EAU mice treated with zebularine or vehicle. The fundus scores and histology scores of EAU mice treated with zebularine (*N* = 16) or vehicle (*N* = 15) on Day 14 are shown in **(B,C)**. Means ± SD are shown ****P < 0.0001.

To investigate the anti-inflammatory effect of zebularine *in vivo*, we treated EAU mice intraperitoneally with zebularine and investigated the changes of intraocular inflammation. Fundus examination was performed every 7 days to evaluate the severity of intraocular inflammation in EAU mice. As shown in Figures 6A,B, the retinal tissue damage started on Day 7 and peaked at Day 14 post immunization. However, zebularine treatment significantly reduced the severity of intraocular inflammation and retinal tissue damage, evidenced by the fundus clinical score reduction from 2.91 ± 0.24 (vehicle treated group) to 0.88 ± 0.16 (zebularine treated group) on Day 14. Importantly, during the 5 weeks when mice received zebularine treatment, we did not observe any body weight changes (Figure 6C). Consistent with the fundus examination result, histological analyses of the retinal tissues from EAU mice on the disease peak Day 14 post immunization revealed that zebularine treatment significantly alleviated intraocular inflammation and retinal tissue damage (Figures 7A–C). Taken together, our data suggest that zebularine significantly reduces intraocular inflammation and retinal damage *in vivo*.

**Figure 8:**
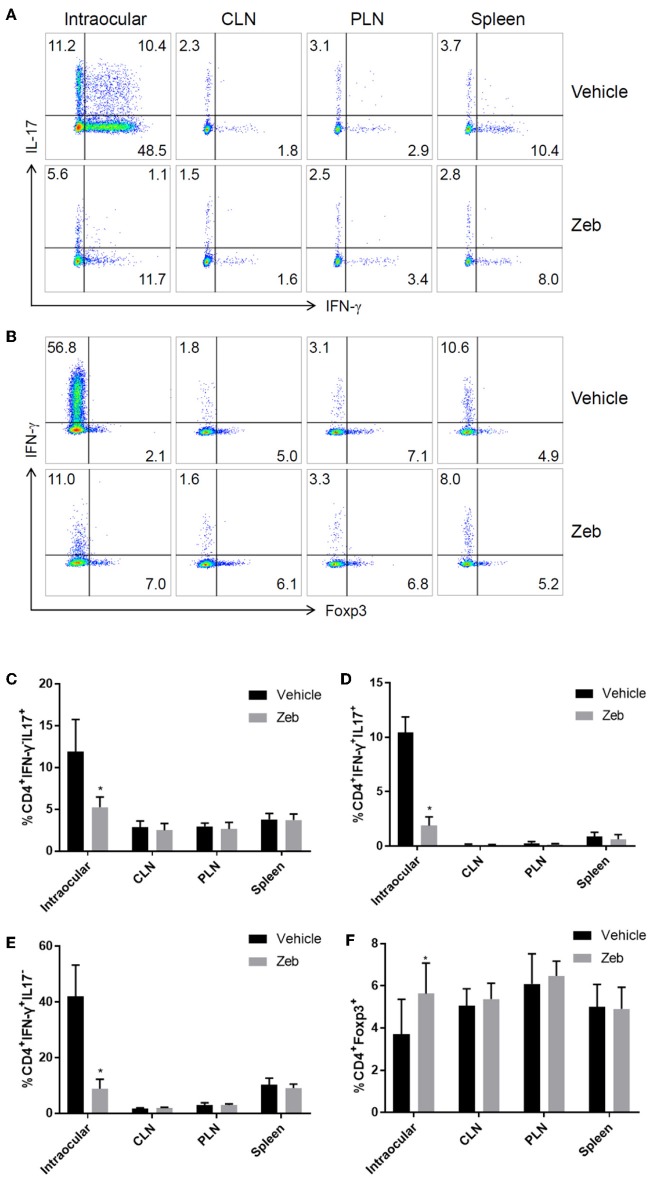
Analysis of CD4^+^ T cells in inflamed eyes, as well as lymph nodes. **(A,B)** The representative FACS analysis of the frequency of IFN-γ^+^, IL-17^+^, and Foxp3^+^ cells in single cell suspensions (pooled from 5 mice) prepared from the eyes, cervical lymph nodes (CLN), peripheral lymph nodes (PLN), and spleens of EAU mice treated with (*N* = 5) or without zebularine (*N* = 5) on Day 14 post-immunization. **(C–F)** Summary of two analysis (total of 10 EAU mice treated with zebularine and 10 without zebularine) of the frequency of IFN-γ^+^, IL-17^+^, and Foxp3^+^ cells in eyes and lymph nodes (each analysis represented the pooled cell population from 5 mice per group). *P < 0.05, paired *T* test (student T).

Since zebularine restrained inflammatory cytokine expression in CD4^+^ T cells *in vitro*, we next prepared single cell suspensions from the eyes, cervical lymph nodes (CLN), peripheral lymph nodes (PLN) and spleen of EAU mice treated with or without zebularine on Day 14 post-immunization and analyzed the changes of IFN-γ, IL-17, and Foxp3 expression. As shown in Figures 8A–E, the frequency of both intraocular CD4^+^IFN-γ^+^ and CD4^+^IL-17^+^ cells was reduced by zebularine, while the frequency of CD4^+^Foxp3^+^ cells was elevated (Figures 8B,F). In contrast, the expression of IFN-γ, IL-17, and Foxp3 was not significantly changed in CLN, PLN, and spleen (Figures 8A–F). Therefore, these data further demonstrate that zebularine controls ocular specific inflammation without significantly altering the systemic CD4^+^ T cell populations.

## Discussion

CD4^+^ T cells mediate systemic and local inflammation in uveitis (22). Our current study suggests that the DNA methylation inhibitor zebularine suppresses the expression of inflammatory cytokines IFN-γ and IL-17 in CD4^+^ T cells *in vitro* and *in vivo*, promotes Foxp3 expression, and alleviates the severity of intraocular inflammation and retinal tissue damage in EAU mice. To our knowledge, this is the first report showing that zebularine is a potential therapeutic agent for uveitis.

Previous studies have demonstrated the clinical utility of DNA methylation inhibitors in oncology, especially in the treatment of myelodysplastic syndrome and acute myeloid leukemia (12). However, it is only recently that these drugs have been evaluated in the context of experimental models of inflammatory disease (14, 15, 23–27). This showed that systemic administration of the two DNA methylation inhibitors azacytidine and decitabine for treating immune-mediated diseases was limited due to their significant cytotoxicity and side-effects (28). In contrast, our data revealed limited cytotoxicity of zebularine on human CD4^+^ T cells *in vitro* and limited systemic toxicity of zebularine in mice *in vivo*, suggesting that zebularine may be a better immunosuppressive agent in comparison to the other DNA methylation inhibitors. Importantly, several reports have identified indoleamine 2,3-dioxygenase (IDO) as the targets of zebularine through which zebularine carried out immunomodulatory functions on cancer and autoimmunity treatments (29–31). Our data on CD4^+^ T cells provided another possible mechanism by which zebularine carried out its immunosuppressive function.

Tbet, RORγt, and Foxp3 are the canonical transcription factors promoting Th1, Th17, and Treg polarization (11). We found that zebularine suppressed the expression of *Tbx21* (encoding Tbet), but promoted the expression of *Foxp3* and *Rorc* (encoding RORγt). Suppression of Tbet in Th1 cells correlated well with the significant reduction of IFN-γ in response to zebularine treatment. Promotion of Foxp3 expression, which is consistent with previous reports that other DNA methylation inhibitors also activate Foxp3 expression in murine models of asthma, EAE and diabetes (14, 24, 32, 33), explained the significant alleviation of intraocular inflammation found in EAU mice. However, upregulation of *Rorc* expression seemed contradictory to the reduction of IL-17 expression by zebularine found both *in vitro* and *in vivo*. Although RORγt is the critical transcription factor promoting IL-17 expression and Th17 differentiation, other transcription factors such as STAT3, IRF4, and BATF are also responsible for the coordinated regulation of IL-17 expression (34). Therefore, the suppression of IL-17 expression by zebularine may depend on a RORγt independent pathway.

Our study demonstrates that zebularine restrains IFN-γ and IL-17 expression and promotes Foxp3 expression in CD4^+^ T cells and may serve as a candidate therapeutic agent for autoimmune uveitis. Further study is warranted to elucidate the molecular mechanisms by which zebularine epigenetically regulates the expression of these key cytokines and transcription factors in T cells.

## Data Availability

The datasets generated for this study are available on request to the corresponding author.

## Ethics Statement

CD4^+^ T cells were obtained from peripheral blood from healthy controls (HCs) following informed consent in accordance with National Health Service Research Ethic Committee approved protocols at the University Hospitals Bristol Foundation Trust, United Kingdom (04/Q2002/84). Written informed consent was obtained from all study participants. All animal experiments were approved by the Institutional Animal Care and Use Committee of Zhongshan Ophthalmic Center. All animal work was performed in compliance with the ARVO Statement for the Use of Animals in Ophthalmic and Vision Research.

## Author Contributions

LW conceived and designed the experiments. YiL, RL, and LW supervised and coordinated the work. YZ, XH, and LS-B performed most experiments and analyzed data. MS, LM, XG, LY, YaL, PB, XW, and JL provided technical assistance and performed experiments. LW drafted and finalized the manuscript.

### Conflict of Interest Statement

The authors declare that the research was conducted in the absence of any commercial or financial relationships that could be construed as a potential conflict of interest.

## References

[1] Jabs DA Nussenblatt RB Rosenbaum JT. Standardization of uveitis nomenclature for reporting clinical data. Results of the First International Workshop. Am J Ophthalmol. (2005) 140:509–16. 10.1016/j.ajo.2005.03.057 16196117 PMC8935739

[2] Forrester JV Klaska IP Yu T Kuffova L. Uveitis in mouse and man. Int Rev Immunol. (2013) 32:76–96. 10.3109/08830185.2012.747524 23360160

[3] Lin P Suhler EB Rosenbaum JT. The future of uveitis treatment. Ophthalmology. (2014) 121:365–76. 10.1016/j.ophtha.2013.08.029 24169255 PMC3913649

[4] Papotto PH Marengo EB Sardinha LR Goldberg AC Rizzo LV. Immunotherapeutic strategies in autoimmune uveitis. Autoimmun Rev. (2014) 13:909–16. 10.1016/j.autrev.2014.05.003 24833504 PMC4181827

[5] Fan H Morand EF. Targeting the side effects of steroid therapy in autoimmune diseases: the role of GILZ. Discov Med. (2012) 13:123–33. 22369971

[6] Noack M Miossec P. Th17 and regulatory T cell balance in autoimmune and inflammatory diseases. Autoimmun Rev. (2014) 13:668–77. 10.1016/j.autrev.2013.12.004 24418308

[7] Eisenstein EM Williams CB. The T(reg)/Th17 cell balance: a new paradigm for autoimmunity. Pediatr Res. (2009) 65(5 Pt 2):26R−31R. 10.1203/PDR.0b013e31819e76c7 19218879

[8] Lee RW Nicholson LB Sen HN Chan CC Wei L Nussenblatt RB. Autoimmune and autoinflammatory mechanisms in uveitis. Semin Immunopathol. (2014) 36:581–94. 10.1007/s00281-014-0433-9 24858699 PMC4186974

[9] Perez VL Caspi RR. Immune mechanisms in inflammatory and degenerative eye disease. Trends Immunol. (2015) 36:354–63. 10.1016/j.it.2015.04.003 25981967 PMC4563859

[10] Wei G Wei L Zhu J Zang C Hu-Li J Yao Z. Global mapping of H3K4me3 and H3K27me3 reveals specificity and plasticity in lineage fate determination of differentiating CD4+ T cells. Immunity. (2009) 30:155–67. 10.1016/j.immuni.2008.12.009 19144320 PMC2722509

[11] O'Shea JJ Paul WE. Mechanisms underlying lineage commitment and plasticity of helper CD4+ T cells. Science. (2010) 327:1098–102. 10.1126/science.1178334 20185720 PMC2997673

[12] Tough DF Tak PP Tarakhovsky A Prinjha RK. Epigenetic drug discovery: breaking through the immune barrier. Nat Rev Drug Discov. (2016) 15:835–53. 10.1038/nrd.2016.185 27765940

[13] Christman JK. 5-Azacytidine and 5-aza-2′-deoxycytidine as inhibitors of DNA methylation: mechanistic studies and their implications for cancer therapy. Oncogene. (2002) 21:5483–95. 10.1038/sj.onc.1205699 12154409

[14] Wu CJ Yang CY Chen YH Chen CM Chen LC Kuo ML. The DNA methylation inhibitor 5-azacytidine increases regulatory T cells and alleviates airway inflammation in ovalbumin-sensitized mice. Int Arch Allergy Immunol. (2013) 160:356–64. 10.1159/000343030 23183158

[15] Chan MW Chang CB Tung CH Sun J Suen JL Wu SF. Low-dose 5-aza-2′-deoxycytidine pretreatment inhibits experimental autoimmune encephalomyelitis by induction of regulatory T cells. Mol Med. (2014) 20:248–56. 10.2119/molmed.2013.00159 24869907 PMC4107100

[16] Billam M Sobolewski MD Davidson NE. Effects of a novel DNA methyltransferase inhibitor zebularine on human breast cancer cells. Breast Cancer Res Treat. (2010) 120:581–92. 10.1007/s10549-009-0420-3 19459041 PMC3901992

[17] Zhu J Paul WE. Heterogeneity and plasticity of T helper cells. Cell Res. (2010) 20:4–12. 10.1038/cr.2009.138 20010916 PMC3494736

[18] Zhang W Li Y Lin J Wan S Gao H Zhang L. Comparison of hematoxylin-eosin staining and methyl violet staining for displaying ghost cells. Eye Sci. (2013) 28:140–3. 24579555

[19] Agarwal RK Silver PB Caspi RR. Rodent models of experimental autoimmune uveitis. Methods Mol Biol. (2012) 900:443–69. 10.1007/978-1-60761-720-4_22 22933083 PMC3810964

[20] Huehn J Polansky JK Hamann A. Epigenetic control of FOXP3 expression: the key to a stable regulatory T-cell lineage? Nat Rev Immunol. (2009) 9:83–9. 10.1038/nri2474 19114986

[21] Morikawa H Sakaguchi S. Genetic and epigenetic basis of Treg cell development and function: from a FoxP3-centered view to an epigenome-defined view of natural Treg cells. Immunol Rev. (2014) 259:192–205. 10.1111/imr.12174 24712467

[22] Luger D Caspi RR. New perspectives on effector mechanisms in uveitis. Semin Immunopathol. (2008) 30:135–43. 10.1007/s00281-008-0108-5 18317764 PMC2756230

[23] Mangano K Fagone P Bendtzen K Meroni PL Quattrocchi C Mammana S. Hypomethylating agent 5-aza-2′-deoxycytidine (DAC) ameliorates multiple sclerosis in mouse models. J Cell Physiol. (2014) 229:1918–25. 10.1002/jcp.24641 24700487

[24] Zheng Q Xu Y Liu Y Zhang B Li X Guo F. Induction of Foxp3 demethylation increases regulatory CD4+CD25+ T cells and prevents the occurrence of diabetes in mice. J Mol Med. (2009) 87:1191–205. 10.1007/s00109-009-0530-8 19841877

[25] Dunn J Qiu H Kim S Jjingo D Hoffman R Kim CW. Flow-dependent epigenetic DNA methylation regulates endothelial gene expression and atherosclerosis. J Clin Invest. (2014) 124:3187–99. 10.1172/JCI74792 24865430 PMC4071393

[26] Cao Q Wang X Jia L Mondal AK Diallo A Hawkins GA. Inhibiting DNA Methylation by 5-Aza-2′-deoxycytidine ameliorates atherosclerosis through suppressing macrophage inflammation. Endocrinology. (2014) 155:4925–38. 10.1210/en.2014-1595 25251587 PMC4239421

[27] Guo H Wang W Zhao N He X Zhu L Jiang X. Inhibiting cardiac allograft rejection with interleukin-35 therapy combined with decitabine treatment in mice. Transpl Immunol. (2013) 29:99–104. 10.1016/j.trim.2013.10.001 24103733

[28] Issa JP Garcia-Manero G Giles FJ Mannari R Thomas D Faderl S. Phase 1 study of low-dose prolonged exposure schedules of the hypomethylating agent 5-aza-2′-deoxycytidine (decitabine) in hematopoietic malignancies. Blood. (2004) 103:1635–40. 10.1182/blood-2003-03-0687 14604977

[29] Liu H Xue ZT Sjögren HO Salford LG Widegren B. Low dose Zebularine treatment enhances immunogenicity of tumor cells. Cancer Lett. (2007) 257:107–15. 10.1016/j.canlet.2007.07.013 17768004

[30] Nittby H Ericsson P Förnvik K Strömblad S Jansson L Xue Z. Zebularine induces long-term survival of pancreatic islet allotransplants in streptozotocin treated diabetic rats. PLoS ONE 8:e71981. 10.1371/journal.pone.0071981 23991016 PMC3753325

[31] Xue ZT Sjögren HO Salford LG Widegren B. An epigenetic mechanism for high, synergistic expression of indoleamine 2,3-dioxygenase 1 (IDO1) by combined treatment with zebularine and IFN-gamma: potential therapeutic use in autoimmune diseases. Mol Immunol. (2012) 51:101–11. 10.1016/j.molimm.2012.01.006 22424783

[32] Kennedy A Schmidt EM Cribbs AP Penn H Amjadi P Syed K. A novel upstream enhancer of FOXP3, sensitive to methylation-induced silencing, exhibits dysregulated methylation in rheumatoid arthritis Treg cells. Eur J Immunol. (2014) 44:2968–78. 10.1002/eji.201444453 25042153

[33] Someya K Nakatsukasa H Ito M Kondo T Tateda KI Akanuma T. Improvement of Foxp3 stability through CNS2 demethylation by TET enzyme induction and activation. Int Immunol. (2017) 29:365–75. 10.1093/intimm/dxx049 29048538 PMC5890887

[34] Ciofani M Madar A Galan C Sellars M Mace K Pauli F. A validated regulatory network for Th17 cell specification. Cell. (2012) 151:289–303. 10.1016/j.cell.2012.09.016 23021777 PMC3503487

