# Supplementary material for: The DNA Methylation Inhibitor Zebularine Controls CD4+ T Cell Mediated Intraocular Inflammation
Source: Front Immunol. 2019 Aug 16;10:1950. doi: 10.3389/fimmu.2019.01950 (PMC6706956; doi:10.3389/fimmu.2019.01950)
Supplement: Supplementary file 1 [file Table_1.docx]

**Supplementary Table 1. Primers for real-time PCR**

| Gene | primer (5'-3') |
| --- | --- |
| *Ifng* F | AGAGGATGGTTTGCATCTGGGTCA |
| *Ifng* R | ACAACGCTATGCAGCTTGTTCGTG |
| *Il17a* F | GCTCCAGAAGGCCCTCAGACT |
| *Il17a* R | CCAGCTTTCCCTCCGCATTGA |
| *Tbx21* F | AGCAAGGACGGCGAATGTT |
| *Tbx21* R | GGGTGGACATATAAGCGGTTC |
| *Rorc* F | GAGGTGTGGGTCTTCTTTGCAGC |
| *Rorc* R | GGAGGGCAGCAAGGACGGCAC |
| *Foxp3* F | CCAGTACTCAGGGCAGTGT |
| *Foxp3* R | GTGGAAGAACTCTGGGAAGG |
| *Gapdh* F | ACCCAGAAGACTGTGGATGG |
| *Gapdh* R | CACATTGGGGGTAGGAACAC |
